# 3D model-assisted instrumentation of the pediatric spine: a technical note

**DOI:** 10.1186/s13018-021-02743-5

**Published:** 2021-10-12

**Authors:** Marko Jug, Matevž Tomaževič, Matej Cimerman

**Affiliations:** Department of Traumatology, University Medical Centre Ljubljana, Zaloška cesta 7, 1000 Ljubljana, Slovenia

**Keywords:** 3D printed model, Virtual preoperative planning, In vitro testing, Pediatric spinal instrumentation

## Abstract

**Background:**

Instrumentation of the pediatric spine is challenging due to anatomical constraints and the absence of specific instrumentation, which may result in iatrogenic injury and implant failure, especially in occipito-cervical constructs. Therefore, preoperative planning and in vitro testing of instrumentation may be necessary.

**Methods:**

In this paper, we present a technical note on the use of 1:1 scale patient-specific 3D printed spinal models for preoperative assessment of feasibility of spinal instrumentation with conventional spinal implants in pediatric spinal pathologies.

**Results:**

The printed 3D models fully matched the intraoperative anatomy and allowed a preoperative confirmation of the feasibility of the planned instrumentation with conventional screws for adult patients. In addition, the possibility of intraoperative model assessment resulted in better intraoperative sense of spinal anatomy and easier freehand screw insertion, thereby reducing the potential for iatrogenic injury. All 3D models were printed at the surgical department at a very low cost, and the direct communication between the surgeon and the dedicated specialist allowed for multiple models or special spinal segments to be printed for more detailed consideration.

**Conclusions:**

Our technical note highlights the critical steps for preoperative virtual planning and in vitro testing of spinal instrumentation on patient-specific 3D printed models at 1:1 scale. The simple and affordable method helps to better visualize pediatric spinal anatomy and confirm the suitability of preplanned conventional spinal instrumentation, thereby reducing X-ray exposure and intraoperative complications in freehand screw insertion without navigation.

## Background

Pediatric spinal instrumentation is complicated by anatomical and technical constraints. Not only do the small vertebrae present a considerable challenge for implant insertion to the treating surgeon, but also the use of adult spinal instrumentation—in the absence of specific instrumentation for the pediatric spine—may result in iatrogenic vertebral injury and implant failure due to fragile spinal elements. The complication rate is particularly notable for patients younger than eight years [1] and in occipito-cervical instrumentation [2]. In addition, spinal surgeons may not perform pediatric spinal instrumentation very often and they may lack the sense of specific spinal anatomy. Therefore, preoperative 3D spinal model printing was suggested to evaluate and improve the sense of spinal anatomy, resulting in improved accuracy of instrumentation [3, 4]. However, preoperative patient-specific 3D model printing may not only prove useful in anatomical considerations as suggested, but may also represent a valuable tool for preoperative assessment of feasibility of the preplanned instrumentation in vitro, thereby additionally reducing the risk of iatrogenic injury and implant failure.

Here we present a technical note on the use of patient-specific 3D printed spinal models in the planning and treatment of pediatric patients with spinal pathologies. The process of preoperative virtual planning and in vitro testing of the preplanned fixation on a 1:1 scale patient-specific 3D printed spinal model for the assessment of feasibility of spinal instrumentation with conventional spinal implants is presented and evaluated. Additionally, the value of intraoperative visualization of spinal anatomy on the in vitro operated 3D model is explored.

## Methods

**Fig. 1:**
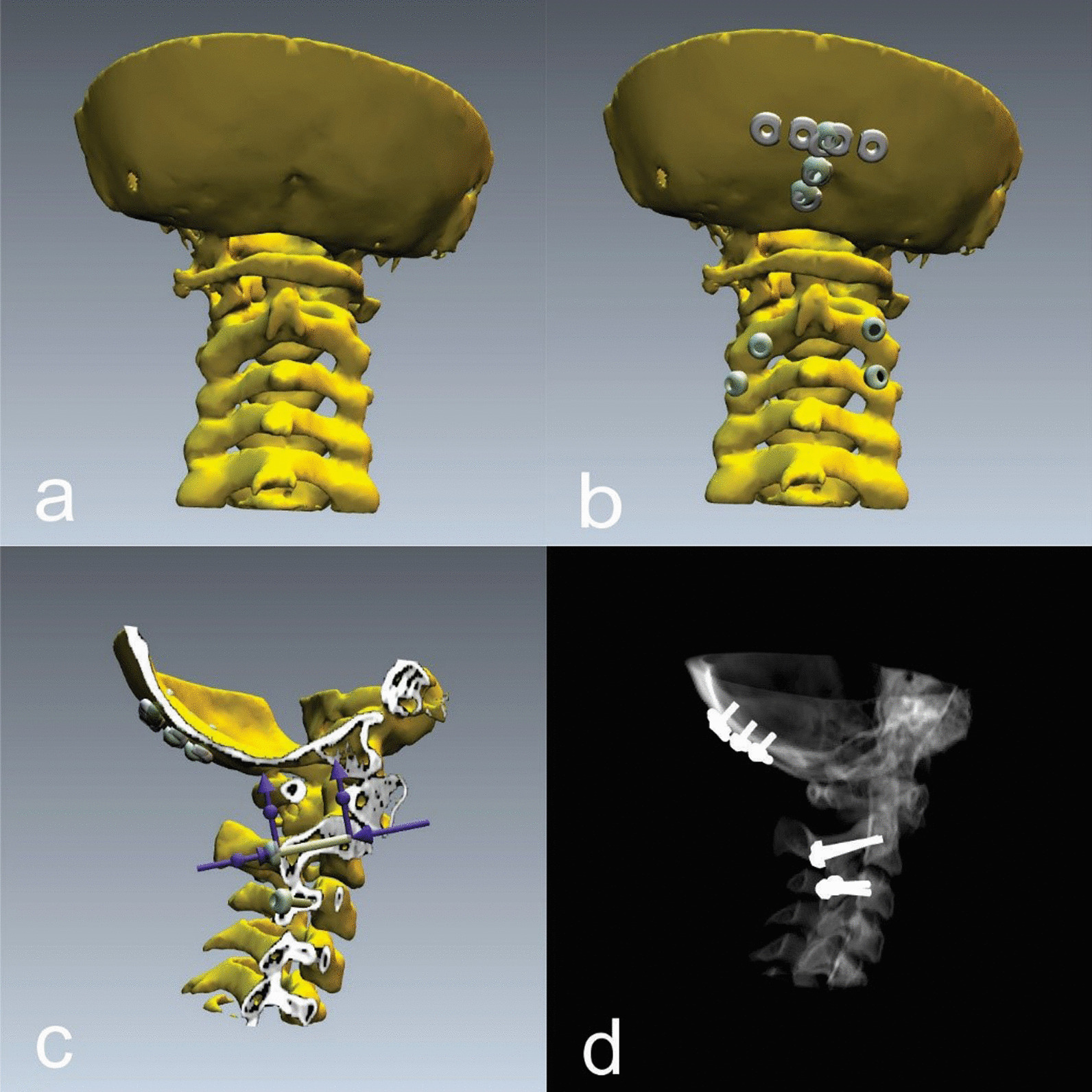
**a** 3D virtual model of spine and occiput anatomy. **b** Simulation of screw positioning. **c** Virtual verification of screws dimensions and the bone stock in the cutaway mode relative to the screw axis. **d** X-ray simulation of the positions of screws

**Fig. 2:**
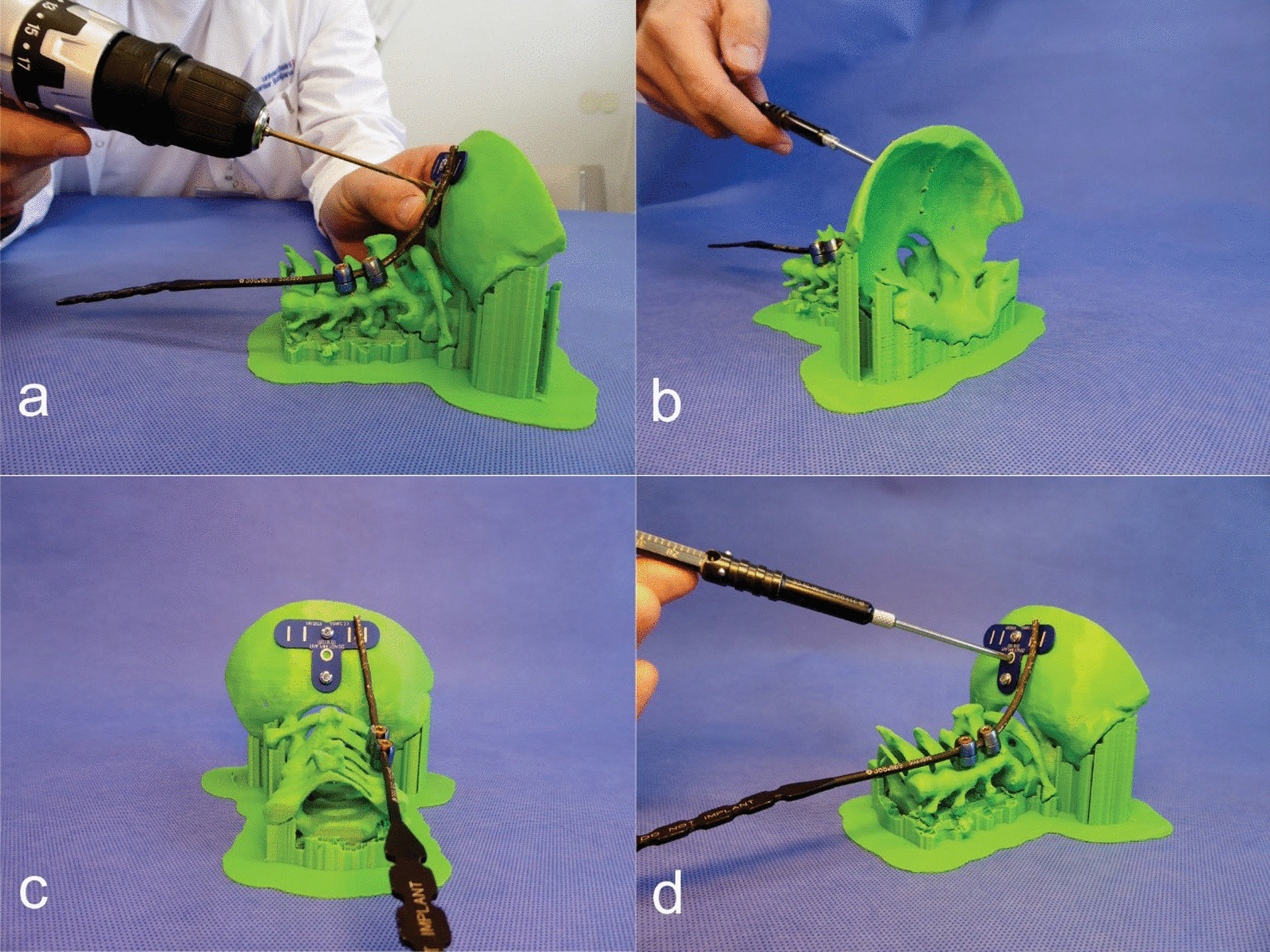
**a** Performing in vitro operation on a 3D printed spine and occiput model. **b** Measuring the occiput region. **c** Instrumentation in place on the 3D model. **d** Verifying that the measurement in the 3D virtual operation was correct

Preoperative 3D planning was performed using the spine EBS software (Ekliptik, Ltd.). DICOM images from standard CT examinations with a slice thickness of 1 mm were used to build the virtual 3D model of the spine in the software. The spinal model was then exported in the .stl file, which was later used for 3D printing. A virtual operation was then performed on the same virtual spinal model with a simulation of available instrumentation (Fig. 1). Instrumentation was simulated using generic 3.5-mm-diameter screws similar to available polyaxial screws for posterior cervical adult spinal instrumentation (Axon, Synthes). After confirming the feasibility of the proposed instrumentation with conventional spinal implants regarding implant size and positioning, a 1:1 scale 3D print was made on a fused deposition modeling desktop printer (Creality CR 10, Creality 3D Technology Co., Ltd.) using a polylactic acid (PLA) filament of 1.75 mm in diameter (AzureFilm, Ltd.) with the shell structure thickness set to 2 mm. The infill had a 20% density in hexagon shape. Preparation for 3D printing was made with shareware software Cura 4.0 (Ultimaker, Ltd.). Three 3D printed spinal models were prepared for each case: one for preoperative anatomical consideration and testing of various instrumentation techniques, one for the final preoperative instrumentation (Fig. 2) and one for intraoperative anatomical consideration (Fig. 4c). The final preoperative instrumentation was performed in vitro using a conventional modular 3.5 mm polyaxial screw system designed for adult posterior cervico-occipital fixation (Synthes, Axon) with the preplanned screw sizes to test the preplanned screw trajectory, positioning and dimensions (Fig. 2). In vitro surgery confirmed the feasibility of the virtual plan regarding screw trajectories and dimensions, which were then recorded for intraoperative use. The 3D model was later used intraoperatively for anatomical consideration, and the virtual X-ray simulation was compared with postoperative X-rays. Written *informed consent was obtained from all participants.*

## Results

### Case 1

**Fig. 3:**
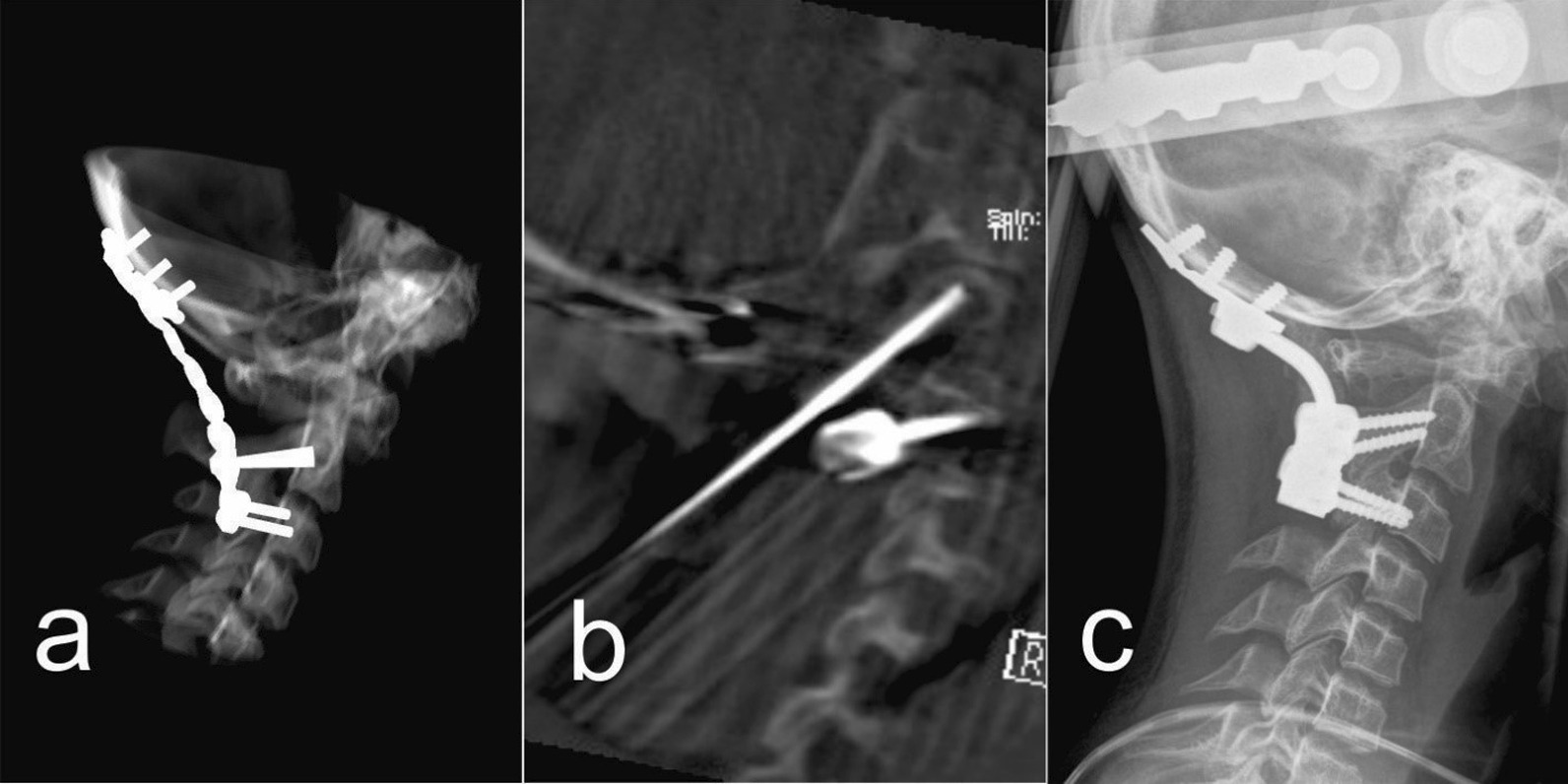
**a** X-ray simulation in preoperative planning software. **b** Intraoperative CT verification. **c** Postoperative control X-ray in HALO jacket

A 12-year-old girl presented with chronic Grisel’s syndrome, a non-traumatic atlantoaxial subluxation resistant to conservative treatment, without neurological deficits. Initially, reduction in the Halo vest was attempted and satisfactory realignment was obtained, after which occipito-cervical fusion was planned. Due to the complex anatomical conditions of the chronic C1–C2 subluxation, the occipito-cervical fixation was first planned in the virtual environment (Fig. 1). The virtual plan suggested a fixation from the occiput to the third cervical vertebra with three 3.5 mm bicortical screws in the sagittal plane of the occiput, isthmic screws at the C2 level and articular mass screws at the C3 spinal level (Fig. 1). The suggested fixation was then tested in vitro on a 3D printed model to confirm screw trajectory, positioning and dimensions using 3.5 mm polyaxial screws designed for posterior cervical spine fixation in adults (Axon, Synthes) (Fig. 2). The 3D spinal model was later used intraoperatively for anatomical consideration. Intraoperative conditions fully matched the virtual plan and the 3D model, allowing the screw placement according to the planned trajectories and dimensions. The possibility to assess the operated model intraoperatively and implant screws as preplanned and tested in vitro significantly improved the sense of spinal anatomy and aided in freehand screw insertion under fluoroscopic control without spinal navigation. Intraoperative CT (Fig. 3b) and postoperative X-ray (Fig. 3c) confirmed the correct implant size and positioning, which was consistent with the virtual plan (Figs. 1, 3a) and the instrumentation used in the 3D model (Fig. 2). Postoperatively the girl retained the Halo vest for one month and started gradual mobilization of the cervical spine after Halo vest removal. Spinal fusion and recovery were uneventful.

### Case 2

**Fig. 4:**
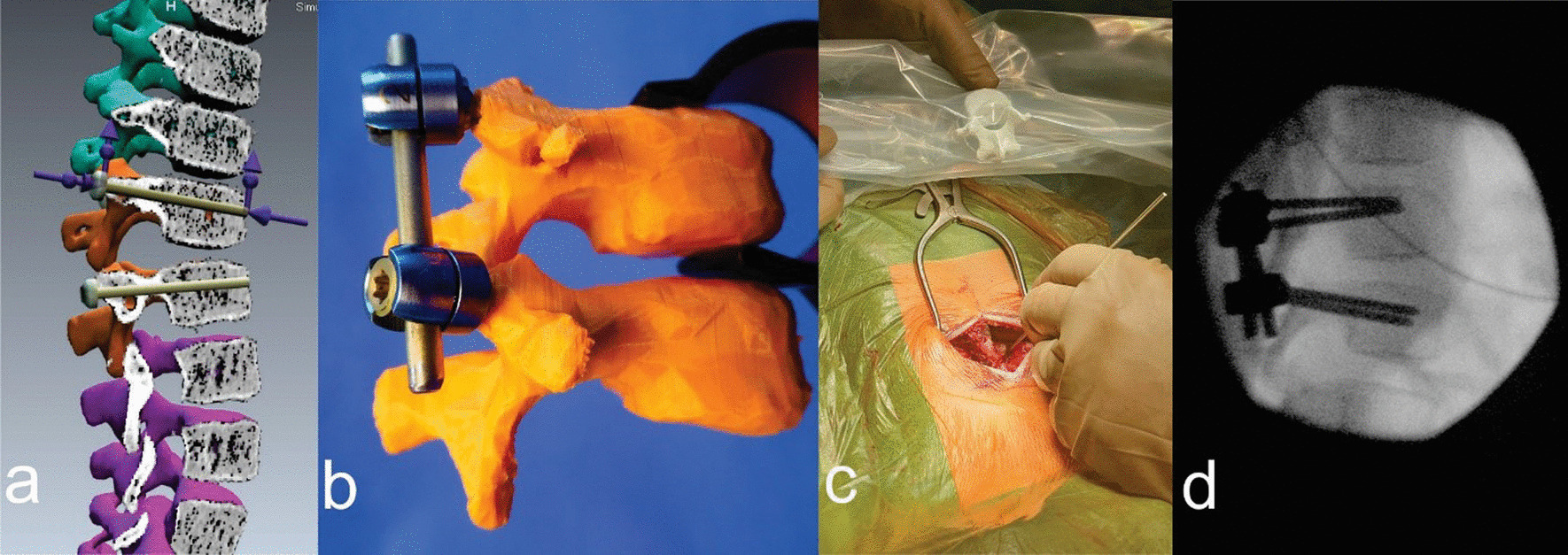
**a** Virtual 3D preoperative planning of the instrumentation. **b** In vitro positioning of the implants. **c** Operation with the 3D model on display. **d** Intraoperative image intensifier control

A 4-year-old girl suffered a distraction ligamentous injury at the thoracic level T11–T12 without neurologic involvement. As the CT showed extremely small pedicles at the T11–T12 level, spinal instrumentation was first assessed as described in the first case (Fig. 4). Virtual and in vitro assessments proved that 3.5 mm × 30 mm polyaxial screws designed for posterior cervical spine fixation in adults (Axon, Synthes) can be used as pedicel screws (Fig. 4a, b). The 3D printed model was additionally used intraoperatively for spinal assessment (Fig. 4c), which allowed freehand screw placement without navigation. The Intraoperative conditions were fully consistent with the virtual and in vitro preplanned model (Fig. 4d), reducing the need for radiography and the risk of iatrogenic injury and implant misplacement or implant failure. Implants were removed after uneventful recovery and spinal fusion six months after injury.

## Discussion

In this technical note, we present the use and efficacy of virtual preoperative planning and preoperative in vitro assessment of fixation techniques in the treatment of spinal pathologies in children. The 3D printed model-assisted spinal instrumentation allowed reliable assessment of instrumentation options prior to surgery, thereby reducing the risk of implant–spine mismatch, screw misplacement and iatrogenic injury to the delicate pediatric spine. In addition, the ability of intraoperative assessment of the preoperated 3D spinal model resulted in an improved sense of spinal anatomy and easier freehand screw insertion with less X-ray exposure. In vitro assessment of feasibility of the fixation allowed conventional spinal implants designed for adults to be used as pedicel and lateral mass screws even in very young patients. It should be noted that the unavailability of specifically designed pediatric spinal instrumentation presents a significant challenge for the treating surgeon. Especially in patients younger than eight years, the small and fragile vertebral elements predispose the spine to iatrogenic injury if conventional instrumentation is used [1, 5]. In addition, spinal surgery in this age group is rare and the surgeon may lack the appropriate experience with instrumentation of such a small spine [1]. A notable complication rate was observed particularly with bicortical occipital screw placement in occipito-cervical constructs [2]. However, intraoperative complications may be reduced with preoperative planning and in vitro testing. Therefore, in our view, preoperative in vitro instrumentation is of great help to the surgeon as it helps him to better identify anatomical features and test the feasibility, dimensions and positioning of the virtually planned fixation. Accordingly, recent studies have shown that preoperative planning and 3D model printing help to improve the accuracy of screw positioning [3, 4]. However, 3D printing in spinal surgery is generally used for anatomical considerations [3, 4, 6–8] or for printing of drill guide templates [9, 10], but not for preoperative in vitro testing of instrumentation. The development of new desktop printers and printing materials certified for use in humans will allow the ideal combination of model and drill guide template printing, adding additional value to 3D printing on surgical wards. On the other hand, higher accuracy of pedicle screw placement has been shown by using computer-guided navigation in pediatric spine surgery [11]. However, due to the very flexible pediatric spine the procedure usually requires additional 3D imaging after intraoperative positioning, resulting in increased radiation exposure to the patient. In addition, the procedure includes increased setup time and costs [11]. On the contrary, the use of 3D models is associated with lower costs and radiation dose, while still offering advantages in freehand screw positioning, as discussed previously. Nevertheless, a 3D model-assisted instrumentation cannot be viewed as a replacement for navigation, but rather as a complementary tool in the surgeon's armamentarium.

For a successful in vitro instrumentation, the 3D print must be of high quality, in the sense that its density must be similar to that of bone and it must allow manipulations, drilling and screw insertion. In our series, PLA filament of 1.75 mm in diameter was used and the infill was set to a 20% density hexagon shape to achieve the lowest contractility after printing, which resulted in a 3D print that closely resembled the virtual model and anatomical conditions. Additionally, the thickness of the shell structure of the print was set to 2 mm for drilling. At least three 3D printed spinal models were created for each case so that different instrumentation techniques could be tested before final preoperative instrumentation and intraoperative anatomical consideration (Fig. 4c). In our series, we used standard pediatric CT protocols with a slice thickness of 1 mm to build the virtual 3D models of the spine. Although a slice thickness of less than 1 mm could result in better model accuracy, with the use of modern software a slice thickness of 1 mm is sufficient to prepare detailed 3D models, suggesting that a larger dose of radiation needed for slice thicknesses of less than 1 mm may not be justified.

The 3D printed models were printed on the department printer at very low expenses with an average printing time of 10 h per model. Although we are fully aware that the printing process could be much faster if professional machines were used, we believe that it is much more beneficial to use this simple printing technique in a surgical department than to rely on outside companies, as no time is wasted on communication with external service providers, the need to transfer patient data and the associated data anonymization is eliminated, the printing may be more easily adapted to the surgeon’s specific needs and multiple copies or only specific segments of the spine may be printed at very low cost.

## Conclusion

Our technical note highlights the critical steps for preoperative virtual planning and in vitro testing of spinal instrumentation on patient-specific 3D printed models at 1:1 scale, which helps to better visualize pediatric spinal anatomy and confirm the suitability of preplanned conventional spinal instrumentation. In addition, the ability to intraoperatively assess the 3D model operated in vitro can be of great help in freehand screw insertion without navigation, thereby reducing X-ray exposure and intraoperative complications at a very low cost. Due to its simplicity and affordability, the use of 3D printing in preoperative planning of pediatric spinal pathologies is strongly recommended.

## Data Availability

Not applicable.
